# High sensitivity of domestic pigs to intravenous infection with HEV

**DOI:** 10.1186/s12917-018-1713-8

**Published:** 2018-12-04

**Authors:** Lisa Dähnert, Martin Eiden, Josephine Schlosser, Christine Fast, Charlotte Schröder, Elke Lange, Albrecht Gröner, Wolfram Schäfer, Martin H. Groschup

**Affiliations:** 1 Institute of Novel and Emerging Infectious Diseases, Friedrich-Loeffler-Institut, Südufer 10, 17493 Greifswald, Insel Riems Germany; 2 Department of Veterinary Medicine, Institute of Immunology, Freie Universität Berlin, Robert-von-Ostertag-Straße 7-13, 14163 Berlin, Germany; 3 Department of Experimental Animal Facilities and Biorisk Management, Friedrich-Loeffler-Institut, 17493 Greifswald, Insel Riems Germany; 4 PathoGuard Consult, Fasanenweg 6, 64342, Seeheim-Jugenheim, Germany; 5 CSL Behring Biotherapies for Life™, P.O. Box 1230, 35002 Marburg, Germany

**Keywords:** HEV, Minimal infectious dose, Swine, In vivo

## Abstract

**Background:**

Hepatitis E virus (HEV) is one major cause of acute clinical hepatitis among humans throughout the world. In industrialized countries an increasing number of autochthonous HEV infections have been identified over the last years triggered by food borne as well as – to a much lower degree – by human to human transmission via blood transfusion. Pigs have been recognised as main reservoir for HEV genotype 3 (HEV-3), and zoonotic transmission to humans through undercooked/raw meat is reported repeatedly. The minimal infectious dose of HEV-3 for pigs is so far unknown.

**Results:**

The minimum infectious dose of HEV-3 in a pig infection model was determined by intravenous inoculation of pigs with a dilution series of a liver homogenate of a HEV infected wild boar. Seroconversion, virus replication and shedding were determined by analysis of blood and faeces samples, collected over a maximum period of 91 days. A dose dependent incubation period was observed in faecal shedding of viruses employing a specific and sensitive PCR method**.** Faecal viral shedding and seroconversion was detected in animals inoculated with dilutions of up to 10^− 7^. This correlates with an intravenously (i.v.) administered infectious dose of only 6.5 copies in 2 ml (corresponding to 24 IU HEV RNA/ml). Furthermore the first detectable shedding of HEV RNA in faeces is clearly dose dependent. Unexpectedly one group infected with a 10^− 4^ dilution exhibited prolonged virus shedding for more than 60 days suggesting a persistent infection.

**Conclusion:**

The results indicate that pigs are highly susceptible to i.v. infection with HEV and that the swine model represents the most sensitive infectivity assay for HEV so far. Considering a minimum infectious dose of 24 IU RNA/ml our findings highlights the potential risk of HEV transmission via blood and blood products.

**Electronic supplementary material:**

The online version of this article (10.1186/s12917-018-1713-8) contains supplementary material, which is available to authorized users.

## Background

Hepatitis E is caused by Hepatitis E virus (HEV) which is a major cause of acute hepatitis throughout the world with a total number of 44,000 HEV-related deaths in 2015 [1]. Hepatitis E virus is a small, quasi-enveloped, single-stranded RNA virus and a member of the *Hepeviridae* family. Novel taxonomic classification consists of the two genera *Piscihepeviru*s and *Orthohepevirus encompassing species A-D.* All mammalian HEV isolates have been attributed to species *Orthohepevirus A* [2] and are further grouped into genotypes 1–8. Although displaying a highly diverse group on molecular level, all genotypes evidently belong to one serotype [3, 4].

Genotype 1 (HEV-1) and 2 (HEV-2) are restricted to humans and are the main cause of endemic outbreaks in developing countries in Asia, Africa and Central America [5, 6]. The transmission of these isolates mainly occurs by the faecal-oral route due to poor sanitation and contaminated water [3].

HEV genotypes 3 (HEV-3) and 4 (HEV-4) dominate in developed countries and are the main source for autochthonous human cases [7]. In Europe, North America, Australia and New Zealand HEV-3 is reported as causative agent for these autochthone HEV infections [8, 9]. In addition, infections with gt4 have been observed in China and Japan [10–12]. Besides humans various wild and domesticated animal species such as wild boar and pigs [13] have been found to carry HEV-3 and HEV-4; HEV-3 has also been detected in deer [14, 15] and rabbits [16, 17]. HEV-3/4 therefore is a zoonosis and pigs as well as wild boar represent the main reservoirs [7, 18]. Domestic pig populations worldwide frequently include viraemic animals and high HEV seroprevalences on herd level [5, 19, 20]. The exact pathogenesis of the HEV infection in pigs has still to be clarified. Naturally the infection occurs by faecal-oral transmission route which has been experimentally shown by a number of studies [21–24], albeit the intravenous infection is used most frequently in experimental challenge studies [22, 24–29]. Infected animals have high viral loads of HEV RNA mainly in the liver accompanied by faecal virus shedding in high concentrations [22, 23, 25, 26, 28]. Remarkably, no clinical symptoms were observed in general [5, 22, 25, 26]. Domestic pigs are a suitable and sensitive model for HEV-3 infection studies since they are also susceptible to human HEV-3 isolates [27].

The HEV-3 transmission from animals to humans via the consumption of infected undercooked/raw meat has been documented in numerous cases [7, 9]. Furthermore, HEV has been detected in processed food products like sausages in Germany [30], Italy [31] and France [9]. Regional distinctions such as raw meat consumption, liver delicacies and close proximity to livestock can have an influence on the exposure towards HEV [8, 32]. Additionally, people with work-related exposure to reservoir animals such as veterinarians, slaughterhouse personal, hunters or stable hands show significantly higher seroprevalences compared to the general population [33, 34].

In general HEV causes a wide range of symptoms, from subclinical to acute hepatitis with icterus up to fulminant hepatic failure [13]: The strictly human pathogenic genotypes HEV-1/2 the main source of endemic outbreaks – cause frequently an acute self-limited hepatitis with icterus and affect in general younger patients [35]. HEV-1 infections in pregnant women are often associated with severe courses, especially in the third trimester [3, 13]. HEV-3 and HEV-4 strains can additionally trigger extrahepatic manifestations but exhibit significantly lower mortality [36]. In contrast to HEV-1, HEV-3 infections can cause chronic and persistent infections mainly in immunocompromised patients [37, 38]. So far the infectivity of HEV is not clearly understood and difficult to determine and due to the lack of a sensitive cell culture system. Since domestic pig are highly susceptible to HEV the porcine model was selected to perform an endpoint titration study based on the application of serial dilutions of a HEV-3 positive liver homogenate. Since swine share many similarities with humans in physiology and immunology the determination of the minimal infectious dose provides important indications also for HEV infectivity in humans.

## Results

### Inoculum

The infection studies were carried out using tenfold dilutions of the inoculum from 10^− 2^ up to 10^− 9^. Corresponding ct-values, copy numbers and IU are summarized in Table 1. 3.7 IU correspond to 1 copy/μl RNA which was calculated from standard curves of both PCR assays used in this study [39] (Additional files 1 and 2). According to this calculation, a 10^− 2^ dilution of the experimentally infected liver contained 9.4 × 10^5^ copies (3.4 × 10^6^ IU) in 2 ml respectively.

**Table 1:** RT-qPCR Values of the inocula after infection

Exp. No.	Dilution	Inoculum				
	Group	CT-Value	cop/μl RNA	cop/ml^t^	cop/dose^t^	IU/dose^t^
1	10–2	25,6	13,010	467.853,40	935.706,80	3.443.401,02
	10–3	28,7	139	49.642,46	99.284,92	365.368,51
	10–4	33,0	5,61	2.003,56	4.007,11	14.746,17
2	10–4	32,2	10	3.607,11	7.214,22	26.548,33
	10–5	35,5	0,91	325,71	651,42	2.397,23
	10–6	no ct	–	–	65,1^a^	239,70*
	10–7	no ct	–	–	6,5^a^	24,00*
	10–8	no ct	–	–	0,65^a^	2,40*
	10–9	no ct	–	–	0,065^a^	0,24*

Viral copy numbers were calculated from CT values determined by RT-qPCR (HEV copies/μl RNA), volume of dose applied was 2 ml, ^*t*^*calculated based on HEV copies/μl RNA (see* Additional files 1 and 2*)*^*a*^*extrapolated values*

### Clinical parameters and pathology

Through-out the whole observation period none of the animals showed a febrile response or any clinical signs consistent with hepatitis. Additionally, no signs typical for viral hepatitis were seen at gross examination after necropsy.

### Virus detection in faeces and serum intra-vitam

**Fig. 1:**
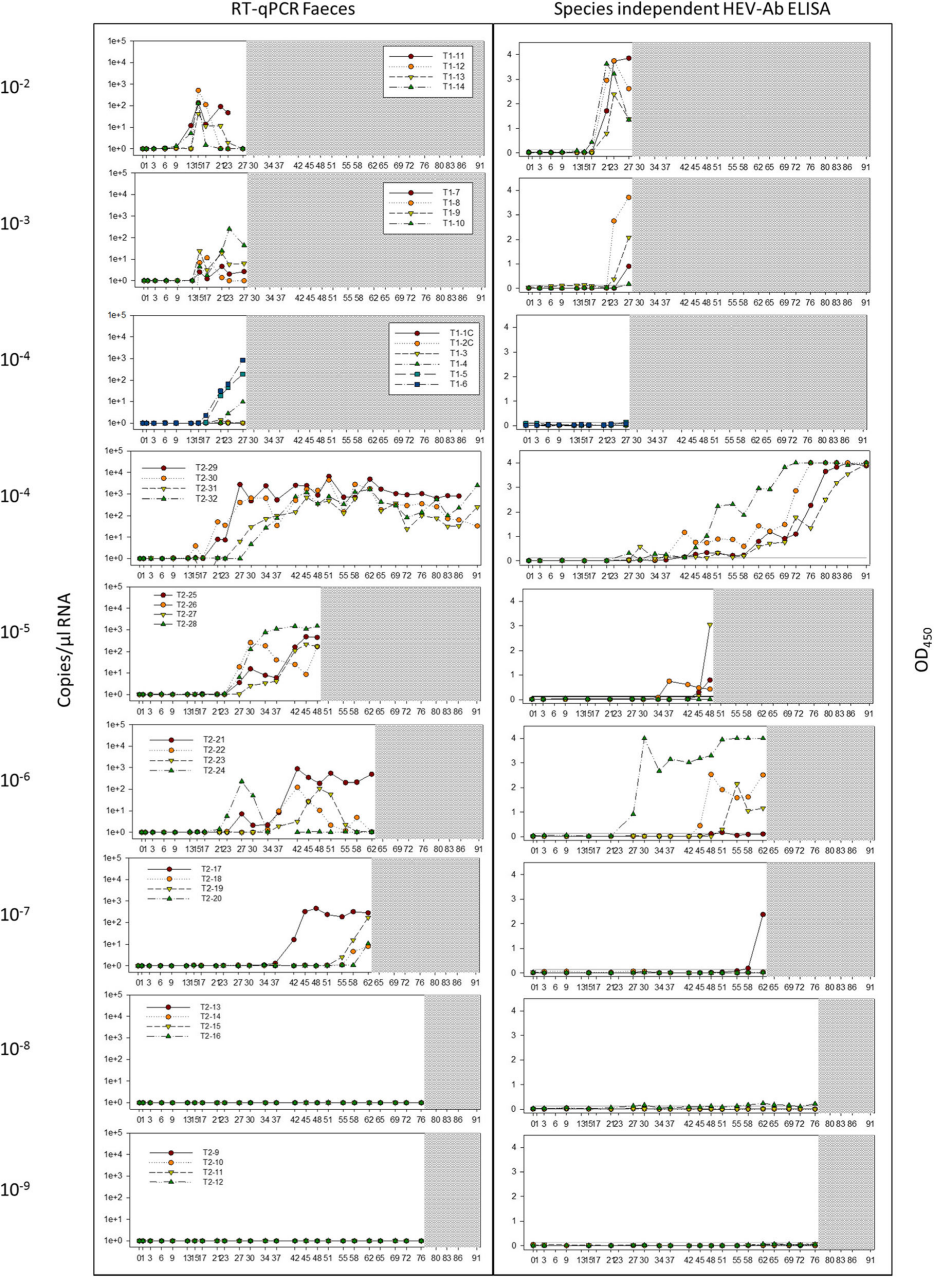
Results of the RT-qPCR from faeces and the species independent HEV-Ab ELISA; the figures display the individual curves of viral RNA in faeces and the detection of total serum antibodies in correlation to days after infection

Viral RNA was detected in the faeces of all groups up to a dilution of 10^− 7^. In the first experimental set up covering homogenate dilutions from 10^− 2^ to 10^− 4^ over an observation period of 27 days (Fig. 1) viral shedding was observed in all three groups. First detection of viral RNA was in the group 10^− 2^ at 9 dpi (pig T1–14) followed by groups 10^− 3^ at 15 dpi and 10^− 4^ at 17 dpi. Due to this delayed onset it was decided to extend the observation period for the second experimental set up for up to 91 days. The second 10^− 4^ group started with viral shedding also at 17dpi, followed by the group 10^− 5^ at 27 dpi. Viruses started to be shed within a time frame of six days for all animals within all groups of the first experiment and these two groups in the second experiment. In the remaining groups inoculated with higher HEV dilutions, a more variable time frame of viral shedding was obvious. In group 10^− 6^, pig T2–24 shed from 21 dpi - 30 dpi. The remaining animals of this group started one by one after 27 dpi, 34 dpi, and 37 dpi. In group 10^− 7^ pigs shed the virus until end of experiment; pig T2–17 started shedding virus at 37 dpi, while the other animals in this group started shedding at 55 dpi, 58 dpi and 62 dpi.

The majority of animals had between 1.3 and 51.7 HEV copies/μl RNA in the10% faecal suspension initially and reached a plateau of about 10^3^ HEV copies/μl RNA eventually. Only individual animals of the 10^− 4^ and the 10^− 5^ group of the second experiment (Fig. 1) occasionally excreted six fold higher viral loads (up to 6.6 × 10^3^ HEV copies/μl RNA) in their faeces.

Viral clearance was detected in group 10^− 2^ in which shedding of HEV RNA in faeces ceased in three out of four pigs (pig T1–12, pig T1–13, T1–14) after a mean of 7 d (±2.7). One individual (pig T1–11) remained positive till the end of the observation period on day 23. Unfortunately on day 27 (necropsy) no faeces was available from this animal for testing. In the 10^− 3^ group only one animal (pig T1–08) cleared the virus before necropsy (27 dpi) 23 days dpi. No viral clearance was observed in the 10^− 4^ group in both experiments even after 91 dpi. For group 10^− 5^ an at least 18d period of faecal shedding was seen with no clearance till the end of the observation period of 49dpi. In group 10^− 6^ virus clearance occurred at very diverse intervals including 11d (pig T2–24), 21d (pig T2–23), 28d (pig T2–22) and more than 35d (pig T2–21), and in group 10^− 7^ the animals shed virus for at least 36d (pig T2–17) or for 7, 4 or 1 days before being euthanized. Neither in the dilution 10^− 8^ nor in the dilution 10^− 9^ signs of virus replication or shedding were observed at all over a period of 76dpi.

The detection of HEV RNA in serum was only sporadic and with low viral amounts (Additional files 2). Detection of viral RNA in serum samples at more than two consecutive sampling time points was only observed for groups 10^− 4^/2 (pig T2–29, pig T2–30, pig T2–31, pig T2–32), 10^− 5^ (pig T2–25, pig T2–27, pig T2–28) and 10^− 7^ (pig T2–17) after 24 dpi, 34 dpi and 58 dpi respectively.

### Infectivity titre of liver

The infectivity in the liver tissue used for the inoculation can be quantified by the calculation of an ID_50_ according to Spearman and Kärber [40, 41] under particular assumptions: Restriction to an observation period of 27 days and in the case of longer incubation times the first positive animal within the group. The calculated titre was 6.3 × 10^5^ ID_50_ per 1 ml liver tissue which represents 4.4 × 10^5^ infectious units/ml. Setting this result in relation to the quantitation of HEV RNA in the liver inoculum 773 IU HEV RNA correspond to one infectious unit (for calculations see Additional file 3).

### Antibody detection

Seroconversion started in all animals after the excretion of viral RNA in faeces. Pigs seroconverted in group 10^− 2^ at 17 dpi, group 10^− 3^ at 23 dpi, group 10^− 4^ at 27 dpi, 10^− 5^ at 37 dpi, 10^− 6^ at 27 dpi and 10^− 7^ at 58 dpi. The interval between detection of HEV in faeces and seroconversion was 7.0 ± 1.0 days for group 10^− 2^, 11 ± 1.7 days for group 10^− 3^, 12.8 ± 12.8 days for group 10^− 4^, 16.0 ± 3.5 days for group 10^− 5^, 13.3 ± 7.2 days for group 10^− 6^, 31.0 ± 0.0 days for group 10^− 7^. In one case (pig T2–32) seroconversion was determined prior to virus detection which can be explained by the oscillation of corresponding OD values around the cut-off at sampling days 27 and 30 followed by a strong increase from day 34 on. HEV antibodies were assessed by the species independent HEV-Ab ELISA detecting total serum antibodies (e.g. IgG, IgM, IgA) in blood (Fig. 1). In the Priocheck HEV Ab porcine ELISA which only detects IgG, seroconversion was generally observed three to ten days later (Additional file 4). An exception was found for animals in the 10^− 7^ group, where seroconversion was detected by both ELISA’s at the same day. No seroconversion was observed in the 10^− 8^ and 10^− 9^ groups.

### Virus detection in organs

All animals of the study were subjected to necropsy and blood, faeces, bile and tissue samples were taken and analysed by RT-qPCR for viral RNA (Tables 2 and 3). This included four different liver loci, the gallbladder, the hepatic lymph nodes, the spleen, the mesenteric lymph nodes and the mandibular lymph nodes. All animals which showed faecal shedding at necropsy were found HEV RNA positive in the bile and at least in two loci of the liver. The viral load of the gallbladder was considerably lower compared to bile itself. In pancreas no viral RNA was detected. In all other organs tested, individuals sporadically showed positive results. Corresponding data are shown in Tables 2 and 3.

**Table 2:** Results of RT-qPCR analysis of selected tissue samples groups 10^− 2^ up to 10^− 4^

		10^−2^				10^−3^				10^−4^						10^− 4^			
		T1–11	T1–12	T1–13	T1–14	T1–07	T1–08	T1–09	T1–10	T1-01c	T1-02c	T1–03	T1–04	T1–05	T1–06	T2–29	T2–30	T2–31	T2–32
		27dpi				27dpi				27dpi						91dpi			
Faeces	Ct	no Ct	no Ct	no Ct	no Ct	32.9	no Ct	31.7	29.0	no Ct	no Ct	no Ct	31.1	27.0	25.0	28.4	34.3	34.2	29.4
	*copy*					*2.7*		*6.3*	*44.4*				*9.6*	*193.2*	*838.7*	*68.8*	*1.3*	*1.4*	*35.0*
Bile	Ct	no Ct	no Ct	no Ct	no Ct	32.9	no Ct	30.7	26.6	no Ct	no Ct	31.2	26.7	25.1	23.0	24.4	31.5	30.1	24.1
	*copy*					*2.6*		*13.2*	*267.0*			*9.5*	*243.2*	*777.6*	*3725.9*	*1007.5*	*8.7*	*21.8*	*1246.9*
Liver 1	Ct	31.6	no Ct	no Ct	no Ct	30.8	no Ct	31.4	27.4	no Ct	no Ct	32.3	31.1	26.1	26.0	24.5	29.5	25.7	22.0
	*copy*	*3.3*				*10.8*		*7.0*	*145.5*			*3.5*	*8.7*	*374.0*	*402.8*	*1884.9*	*38.6*	*727.6*	*13,142.2*
Liver 2	Ct	32.8	no Ct	no Ct	no Ct	30.9	no Ct	31.0	27.8	24.9	no Ct	33.1	30.4	27.0	26.4	23.2	30.0	25.9	25.4
	*copy*	*1.5*				*10.6*		*9.9*	*108.0*	*426.1*		*2.0*	*15.4*	*194.4*	*295.9*	*5187.7*	*25.0*	*637.3*	*898.7*
Liver 3	Ct	32.9	25.2	no Ct	no Ct	29.5	no Ct	31.5	27.5	no Ct	no Ct	27.8	26.2	25.7	24.0	24.3	29.6	29.4	31.6
	*copy*	*1.3*	*291.6*			*28.5*		*6.7*	*136.7*			*103.0*	*361.7*	*505.4*	*1753.2*	*2202.3*	*14.7*	*40.8*	*7.3*
Liver 4	Ct	30.9	no Ct	33.1	no Ct	32.5	no Ct	32.6	26.8	no Ct	no Ct	no Ct	32.5	25.4	25.5	24.2	29.2	27.4	30.2
	*copy*	*5.4*		*1.2*		*3.1*		*3.0*	*223.5*				*3.0*	*617.8*	*585.8*	*2354.4*	*46.2*	*191.2*	*21.5*
Gall bladder	Ct	no Ct	no Ct	no Ct	no Ct	31.0	no Ct	32.5	28.2	no Ct	no Ct	32.2	31.1	28.7	25.8	30.0	no Ct	no Ct	32.1
	*copy*					*9.5*		*3.2*	*76.4*			*3.9*	*8.9*	*52.6*	*485.5*	*25.2*			*5.1*
Lnn. Hepat	Ct	no Ct	no Ct	no Ct	no Ct	no Ct	no Ct	33.4	no Ct	no Ct	no Ct	no Ct	no Ct	no Ct	32.1	31.9	no Ct	31.8	32.2
	*copy*							*1.6*							*4.1*	*5.6*		*6.3*	*4.7*
Spleen	Ct	no Ct	no Ct	no Ct	29.1	no Ct	no Ct	no Ct	no Ct	no Ct	no Ct	no Ct	no Ct	33.8	32.1	33.1	no Ct	32.8	32.2
	*copy*				*18.9*									*1.2*	*4.1*	*2.3*		*2.9*	*4.5*
Pancreas	Ct	no Ct	no Ct	no Ct	no Ct	no Ct	no Ct	no Ct	no Ct	no Ct	no Ct	no Ct	no Ct	no Ct	no Ct	no Ct	no Ct	no Ct	no Ct
	*copy*																		
Ln mes	Ct	no Ct	no Ct	no Ct	no Ct	no Ct	no Ct	no Ct	no Ct	no Ct	no Ct	no Ct	no Ct	no Ct	no Ct	30.6	32.8	no Ct	32.1
	*copy*															*16.4*	*2.8*		*4.9*
Ln mand	Ct	no Ct	no Ct	no Ct	no Ct	no Ct	no Ct	no Ct	no Ct	no Ct	no Ct	no Ct	no Ct	no Ct	no Ct	no Ct	no Ct	33.4	no Ct
	*copy*																	*1.8*	

No Ct ≥34.0 (=negative). Viral copy numbers were calculated from CT values determined by RT-qPCR (HEV copies/μl RNA). LN = lymph node

**Table 3:** Results of RT-qPCR analysis of selected tissue samples groups 10^−5^ up to 10^−9^

		10^−5^				10^−6^				10^−7^				10^−8^				10^−9^			
		T2–25	T2–26	T2–27	T2–28	T2–21	T2–22	T2–23	T2–24	T2–17	T2–18	T2–19	T2–20	T2–13	T2–14	T2–15	T2–16	T2–09	T2–10	T2–11	T2–12
		49dpi				63dpi				63dpi				77dpi				77dpi			
Faeces	Ct	n.s.	29.5	26.9	23.9	27.1	no Ct	no Ct	no Ct	26.3	32.3	27.1	32.3	no Ct	no Ct	no Ct	no Ct	no Ct	no Ct	no Ct	no Ct
	*copy*	*n.s.*	*33.1*	*249.4*	*2473.3*	*163.2*				*283.7*	*3.5*	*163.0*	*3.6*								
Bile	Ct	20.3	21.9	20.8	20.2	27.1	no Ct	no Ct	no Ct	24.3	27.5	24.6	25.1	no Ct	no Ct	no Ct	no Ct	no Ct	no Ct	no Ct	no Ct
	*copy*	*36,698.1*	*10,918.1*	*25,355.3*	*39,844.7*	*160.0*				*1269.3*	*124.9*	*1006.4*	*700.5*								
Liver 1	Ct	23.5	23.7	29.4	23.8	23.3	no Ct	no Ct	no Ct	24.4	30.3	no Ct	30.1	no Ct	no Ct	no Ct	no Ct	no Ct	no Ct	no Ct	no Ct
	*copy*	*4016.9*	*3422.8*	*41.9*	*3226.2*	*1572.7*				*686.2*	*7.0*		*7.8*								
Liver 2	Ct	23.0	23.5	28.4	25.2	26.0	no Ct	no Ct	no Ct	23.5	30.6	no Ct	32.2	no Ct	no Ct	no Ct	no Ct	no Ct	no Ct	no Ct	no Ct
	*copy*	*5946.9*	*4025.0*	*86.6*	*1115.6*	*203.2*				*1383.5*	*5.2*		*1.6*								
Liver 3	Ct	25.3	23.1	29.1	25.2	24.7	no Ct	no Ct	no Ct	23.2	28.0	25.9	no Ct	no Ct	no Ct	no Ct	no Ct	no Ct	no Ct	no Ct	no Ct
	*copy*	*1017.0*	*5475.4*	*50.6*	*1120.3*	*534.1*				*1704.9*	*40.2*	*219.8*									
Liver 4	Ct	24.3	22.3	28.2	26.4	22.2	no Ct	no Ct	no Ct	24.1	29.7	26.9	30.3	no Ct	no Ct	no Ct	no Ct	no Ct	no Ct	no Ct	no Ct
	*copy*	*2191.1*	*10,005.2*	*103.4*	*423.1*	*3978.3*				*882.4*	*11.3*	*99.3*	*7.0*								
Gall bladder	Ct	29.5	30.6	28.1	25.9	29.4	no Ct	no Ct	no Ct	26.2	29.9	24.9	29.1	no Ct	no Ct	no Ct	no Ct	no Ct	no Ct	no Ct	no Ct
	*copy*	*39.0*	*15.5*	*110.0*	*634.2*	*14.3*				*171.0*	*9.5*	*478.6*	*17.0*								
Lnn. Hepat	Ct	31.4	29.2	32.6	31.2	30.1	no Ct	no Ct	no Ct	30.9	no Ct	31.5	no Ct	no Ct	no Ct	no Ct	no Ct	no Ct	no Ct	no Ct	no Ct
	*copy*	*8.5*	*45.9*	*3.3*	*10.0*	*7.8*				*4.3*		*2.6*									
Spleen	Ct	no Ct	29.3	33.4	31.9	29.1	no Ct	no Ct	no Ct	28.1	no Ct	no Ct	no Ct	no Ct	no Ct	no Ct	no Ct	no Ct	no Ct	no Ct	no Ct
	*copy*		*43.9*	*1.8*	*5.7*	*17.4*				*39.0*											
Pancreas	Ct	no Ct	no Ct	no Ct	no Ct	no Ct	no Ct	no Ct	no Ct	no Ct	no Ct	no Ct	no Ct	no Ct	no Ct	no Ct	no Ct	no Ct	no Ct	no Ct	no Ct
	*copy*																				
Ln mes	Ct	33.2	32.3	no Ct	no Ct	27.2	no Ct	no Ct	no Ct	30.2	no Ct	no Ct	no Ct	no Ct	no Ct	no Ct	no Ct	no Ct	no Ct	no Ct	no Ct
	*copy*	*2.1*	*4.4*			*74.5*				*7.4*											
Ln mand	Ct	no Ct	33.2	no Ct	31.4	31.3	no Ct	no Ct	no Ct	no Ct	no Ct	no Ct	no Ct	no Ct	no Ct	no Ct	no Ct	no Ct	no Ct	no Ct	no Ct
	*copy*		*2.0*		*8.2*	*3.2*															

No Ct ≥34.0 (=negative). Viral copy numbers were calculated from CT values determined by RT-qPCR (HEV copies/μl RNA). LN = lymph node

### Sequencing

The sequence of the hypervariable region of the inoculum (sequence and reference sequence KP294371 are shown in Additional file 5) was analysed by nested RT-PCR to monitor possible nucleotide exchanges after replication and passage through infected pigs. The original sequence displays a characteristic C/T wobble sequence at position 2200. Likewise virus sequences from isolates from pigs of dilution groups 10^− 2^, 10^− 3^, 10^− 4^, 10^− 5^, 10^− 6^ and 10^− 7^ displayed C/T polymorphisms at this site. In addition to the nucleotide 2200 polymorphism one isolate (derived from animal (T2–28)) had a C to T exchange at position 2322. The alignment was done with a previously published full genome (KP294371). The wobble sequence translates into a proline/serine exchange. The point mutation in animal T2–28 was silent.

### Horizontal controls

*The two horizontal controls (T1-01c, T1-02c) showed no clinical or molecular evidence for a HEV infection and faecal samples were always negative for HEV RNA. However, one liver lobe of pig T1-01c gave a positive RT-qPCR result (shown in* Table *2**).*

## Discussion

An increasing number of human hepatitis E genotype 3 (HEV-3) infections has been observed in developed countries in the last decade. This is accompanied by a substantial proportion of HEV RNA positive blood products and a growing number of transfusion transmitted HEV infections [8]. The high incidence represents a major health concern especially for immunosuppressed patients or people with pre-existing liver diseases. This raises the question of the minimal infectious dose for intravenously applied HEV-3. For this purpose - based on the similarities of pig and human physiology and the assumption of pigs as reliable animal models for human HEV infection, a comprehensive dose-titration study was carried out in domestic pigs to determine the minimum infectious dose. The titration was based on serial dilutions of a HEV RNA positive liver homogenate of an experimentally infected wild boar [23]. It ranged from a 10^− 2^ dilution up to a 10^− 9^ dilution and was applied into groups of 4 pigs each. Seroconversion and viral shedding was observed up to a 10^− 7^ dilution which corresponds to a minimal infection dose of 6.5 copies in 2 ml total volume (according to 24 IU HEV RNA) for swine. In contrast to pigs, the infectious dose for humans is reportedly significantly higher: the transfusion of a platelet concentrate with residual plasma containing 7056–8892 IU HEV RNA was infectious [59]. In another study, the minimal infectious dose of HEV through transfusion was 3.6 × 10^4^ IU [42]. A minimal infectious dose for humans in the order of 10,000 IU HEV RNA was also reported by *F. Rossi*, IPFA [43]. However, a direct correlation is not possible, since our pig study evaluated liver derived HEV in contrast to human blood (plasma) products. The reduced infectivity of HEV positive plasma samples has been recently demonstrated in human liver chimeric mice [44]. In monkeys, intravenous HEV infection studies were performed as well, however generally using inocula from faecal samples. The used dosages encompassed titers of 6.4 log_10_ copies/ml of a HEV-3 isolate [45] as well as 2.45 × 10^5^ IU/ml for a HEV-4 and 7.51 × 10^5^ IU/ml for a HEV-1 isolate [46]. Similar dosages were used in a recent study [47] using different HEV-1 isolates (3.5–6.4 log_10_ IU/ml) and HEV-3 isolates (2.5–9.5 log 10 IU/ml). In each case cynomolgus monkeys could be productively infected.

All infected animals remained asymptomatic and showed no signs of illness such as fever, reduced alertness nor weight loss throughout the experiment which is in line with previous studies in domestic pigs [24, 25, 28]. So far, only singular cases of icterus in HEV infected pigs [27] as well as the elevation of liver specific enzymes in infected wild boar [23] have been reported. This study established faeces as appropriate source to determine the course of virus replication in pigs via RT-qPCR due to weak and sporadic detection of viral RNA in serum with a general lower viral load. This is in accordance with previous reports [22, 48]. It is of special significance that the calculated copy number of 5 copies /ml in the 10^− 7^ dilution lies below the limit of detection of both PCR assays but is sufficient to infect pigs by the intravenous route. This is in accordance with a previous pig titration study where infectivity was observed below the RT-PCR detection limit [28]. In addition a clear correlation between applied dosage and incubation period was determined: A 10^− 2^ dilution of the inoculum induced viral shedding at 9 dpi, followed by virus shedding at 15 dpi (10^− 3^ dilution), 17 dpi (10^− 4^ dilution) and 27 dpi (the 10^− 5^ dilution) in at least one pig per group, on average 30 dpi (10^− 6^ dilution) and on average 51 days (10^− 7^ dilution). The subsequent dilutions also induce growing individual differences in onset of virus shedding varying from 6 to 9 days (10^− 4^ group), 10–18 days (10^− 6^ group) up to 16 days (10^− 7^ group). The increasing extension of incubation period raises the question regarding replication site and persistence of the virus. Further dilutions (10^− 8^, 10^− 9^) of the inoculum induced no HEV infection. In contrast, in the aforementioned study none of the animals inoculated with dilutions below 10^− 4^ were infected [28]. However a direct comparison of both studies was not possible, since the viral load was determined by Genomic Equivalents (GE/ml) and due to the use of faecal material for intravenous inoculation. Both sources are able to induce virus replication as reported in multiple studies. A comparison of these studies regarding onset and duration of virus shedding and seroconversion is difficult because applied doses are incomparable and inoculum sources were different: After i.v. application of HEV positive faeces (10% suspension) virus detection in faeces started 3 dpi [25, 26] or 7–14 dpi [28] and took 1–3 weeks. Using inoculum from a HEV positive pig derived bile led to virus shedding from 3 dpi on [22]. Using high titre liver homogenates i.v. infected pigs started to excrete virus between 2 and 7 days and continued 12–52 days [24, 29]. In another study faecal RNA samples of infected pigs were detected at day 9 to 19 and faecal HEV excretion lasted 21–30 days [21]. Interestingly both sources harbour significant differences because blood as well as hepatocyte/liver derived HEV particles are enveloped and covered with cellular membranes in contrast to non-enveloped HEV from bile or faeces [49–51]. This envelope has been also detected in cell-culture produced HEV particles [50, 52] and appears to modulate infectivity in vitro as well as in vivo. The generation of quasi-enveloped HEV particles by hepatocytes and hepatoma cell lines [51] strongly indicates that HEV from liver homogenate harbors an envelope as well, but experimental data are still lacking. Cell culture derived enveloped HEV revealed an almost tenfold lower infectivity compared to non-enveloped faeces derived HEV in cell culture [53], which may be a consequence of inefficient cell attachment [54]. However, the findings are complicated by the fact, that human liver homogenate as well as faeces from HEV positive patients is capable to infect humanized chimeric mice in contrast to human plasma or cell-culture derived HEV [55]. Therefore the question how biochemical properties of HEV particles modulate infectivity needs further investigation.

A presumably faecal-oral transmission of HEV was observed by one of the horizontal controls (pig T1-01c) within the 10^− 4^ group. The animal exhibited a HEV positive liver as determined by necropsy at 27dpi. The first onset of viral shedding in this group was on 17dpi from pig T1–06 which leads to a maximum incubation period of 10 days. The reported periods between oral intake and HEV particles shed in faeces can range from 7.2d [24] up to 22d [22] and possibly even longer.

Antibody response and seroconversion were assessed by two ELISAs with different specifications: The AXIOM ELISA was a multispecies test for detecting total serum antibodies against HEV (including IgM, IgA and IgG) whereas the PrioCheck ELISA only detected porcine HEV specific IgG antibodies. Specific porcine IgM ELISAs were not available. In general the serum samples were confirmed positive in the AXIOM ELISA three to ten days before the porcine specific IgG ELISA which is consistent with a primary IgM related immune response followed by IgG response. However, no reliable conclusions can be drawn about the specific occurrence of IgM or IgA or of a mixture of both during the primary immune response. In any case IgA antibodies play an important role in the human immune response against HEV [55, 56].

Virus clearance could be observed in different groups examined: Three animals in group 10^− 2^, one individual from 10^− 3^ and three from the 10^− 6^ dilution group cleared the infection within 7d (±2,7d), 8d and 9d up to 27d respectively. All remaining animals were subjected to necropsy prior to a possible virus clearance. All animals of the second 10^− 4^group displayed a prolonged infection where HEV RNA was detectable in faeces for more than 62 days. Necropsy samples of this group harboured high viral load in bile and liver samples. The 10^− 5^ group exhibited faecal viral shedding for 22d and individual animals from group 10^− 6^ and 10^− 7^ group for 35d and 36d, respectively. Again, all animals showed high viral loads in bile and liver at necropsy. In general, HEV is considered to be a transient infection and viral clearance usually occurs within three weeks after first occurrence in faeces [22, 24]. The long-term viral shedding for more than 32 up to 62 days is therefore uncommon and possibly indicates a chronic or persistent infection. Only rare data from similar studies are available: HEV shedding was significantly increased by co-infection with Porcine Reproductive and Respiratory Syndrome Virus (PRRSV) and extended from 9.7 to 48.6 days [57]. However, all animals in this study had been pre-tested to be negative for PRRSV as well as Porcine circovirus 2 (PCV2). This analysis was continued with faeces samples from the second 10^− 4^ group encompassing the whole observation period, to exclude a newly or internal acquired PRRSV and PCV2 infection. Again all samples were negative for both viruses. All animals showing prolonged viral shedding belonged to the dilutions 10^− 4^ or higher, indicating a correlation between inoculated virus load and prolonged viral shedding. The 10–4 g.

Another research study showed extended virus shedding in wild boars for more than 16 weeks [48]. Interestingly the animals showed high antibody titers as well, with apparently no influence on virus shedding and replication. The presence of neutralising antibodies within the context of a HEV infection has been shown in vivo [58, 59] and in vitro [58–60]. Other factors such as age and individual immune status should be taken into account as well.

It may be of interest that in humans various cases of chronic HEV-3 infections were reported in immunosuppressed patients [61]. For all other genotypes such a correlation has not been made so far.

## Conclusion

In conclusion, this study determined an extremely low minimum infectious dose of HEV to elicit a productive infection in pigs. Additionally our work demonstrated a dose dependent incubation time in pig resulting in a delayed onset of virus replication and shedding. Moreover the study emphasises the potential risk of HEV transmission in humans via blood and blood products. Finally further investigations should be undertaken to determine and compare the infectivity through oral transmission.

## Methods

### Inoculum

The inoculum used in this study was prepared from a highly HEV RNA positive liver of an experimentally infected wild boar from a previous HEV-3 infection study [23].

20 g of liver tissue was grounded with mortar, pestle and sterile sea sand. The homogenate was then diluted 1:5 with sterile 1x phosphate buffered saline (1xPBS) to obtain a 20% dilution and centrifuged at 4400 g for 15 min at 4 °C. The supernatant was eventually sterile-filtered through 0.22 μm MILLEX-GP Syringe Filter Unit (Millipore, Ireland), aliquoted and stored at − 80 °C. The inoculum was titrated with the Logarithmic dilutions in sterile 1xPBS starting from a 10^− 2^ dilution (referring to the original liver tissue) to 10^− 9^. The 10^− 2^ dilution was sterile filtered prior to inoculation and further dilutions. The corresponding inocula were prepared shortly before inoculation and kept on ice until use and administered intravenously (i.v.) to 4 animals/group.

### Experimental design

The competent authority of the Federal State of Mecklenburg Western-Pomerania approved all described animal experiments based on European Directive 2010/63/EU and associated national regulation (reference number in Germany LALLF M-V/TSD/7221.3–2.1.014/10, LALLF M-V/TSD/7221.3–2.1-017/13).

For this study 38 domestic pigs (Large White breed) from a commercial breeder (animal husbandry, 18,196 Dummerstorf, Germany) were acquired and housed under containment level 3** conditions. Healthy animals of compatible sizes and ages were allocated randomly to groups by animal technicians. Social incompatibilities were taken into account in few instances for animal welfare reasons. As the study was a titration experiment with a clear cut yes/no readout, the number of animals per group was set as described together with a biostatistician. The experimental design was part of the individualized animal experimental license (reference number in Germany LALLF M-V/TSD/7221.3–2.1.014/10, LALLF M-V/TSD/7221.3–2.1-017/13 according to the Germany animal welfare law (§7 paragraph 1 phrase 2 TierSchG) which was applied for and given after scientific and ethical assessment by an independent advisory board. After entry, the animals were divided into the experimental groups and held in quarantine for 2 weeks. Individuals of each group were housed together whereas each group was held in a separate stable unit. Complete change of clothes was mandatory before entry of each of the rooms. All animals were pretested for HEV and yielded negative PCR findings in faeces and serum as well negative HEV ELISA results. Due to facility limitations the study was divided in two experiments. The groups were randomly formed consisting of 4 animals each as described in Table 4. Two horizontal transmission control animals were included and kept with the 10^− 4^ group. A blinded experimental and analysis design was not possible as researchers and animal technicians were supposed to carry out husbandry and experimental work in a defined way in order to mitigate (cross-) contamination risks: every day animal manipulations started in the low dose challenged animals, followed by intermediate dose challenged pigs and in high-dose animals eventually. Moreover, as only standardized quantitative data were obtained (e.g. body temperatures, qPCR (Ct-values and copy numbers) and ELISA derived data), blinding was not considered necessary.

**Table 4:** Experimental set up for the titration of highly HEV positive liver tissue in log steps in the porcine model

Exp. No.	Dilution = Group	Animal identity	Animals		Sex	Age at inoculation	Observation period
			infected	horizontal control		in weeks	
1	10^−2^	Pig T1–11, −12, −13, −14	4	–	f/m	10	27dpi
	10^−3^	Pig T1–07, −08, −09, −10,	4	–	f/m	10	27dpi
	10^−4^	Pig T1-01c -02c,-03, −04, −05, −06	4	2	f/m	10	27dpi
2	10^−4^	Pig T2–29, −30, −31, −32	4	–	m	11	91dpi
	10^−5^	Pig T2–25, −26, −27, −28	4	–	f/m	11	49dpi
	10^−6^	Pig T2–21, −22, −23, −24	4	–	f	11	63dpi
	10^−7^	Pig T2–17, −18, −19, −20	4	–	f/m	11	63dpi
	10^−8^	Pig T2–13, −14, −15, −16	4	–	m	11	77dpi
	10^−9^	Pig T2–09, −10, −11, −12	4	–	f	11	77dpi

The dilution step of the homogenate as assigned the group designation, f-female, m-male, dpi-days post infection, −- none

After at least two weeks of acclimatisation negative HEV RNA and Anti-HEV-antibody results were reconfirmed by Rt-qPCR and ELISA. Additionally samples were examined by a novel multiplex PCR [62] (Results not shown) to exclude an influence of co-infection with other viral diseases (PRRSV, PCV2). The inoculation was done with 2.0 ml inoculum intravenously given into the *Vena cava cranialis*.

Animal behaviour and rectal body temperatures were checked daily. As described in previous studies body temperatures over 40 °C for at least two consecutive days were considered a febrile response [23, 48]. Depression, diarrhoea, vomitus, icterus, ascites and neurological symptoms were considered signs of acute hepatitis and would have led to an immediate removal and euthanasia of the animal.

During the experiments blood and faecal samples were taken regularly every two to three days. Blood was allowed to clot for 30 min at room temperature and then centrifuged at 2300 g for 12 min. Serum was then collected, aliquoted and stored at − 20 °C. From the faecal samples a 10% faecal suspension was made using 0.89% NaCl-solution. After vortexing and centrifugation (4400 g, 4 °C, 20 min) the supernatant was sterile filtrated using a sterile 0.22 μm MILLEX-GP Syringe Filter Unit (Millipore, Ireland) and stored at − 20 °C. This solution was the starting point for RNA extraction.

At the end of the observation period all animals were slaughtered (electro stunning followed by exsanguination). Animals were euthanized by a veterinarian following EU and German animal welfare regulations and carcasses necropsied by a trained veterinary pathologist assisted by a necropsy technician. Necropsies were performed and samples from blood, faeces and bile as well as different tissue samples were taken for RNA extraction and stored at − 20 °C. Retrieved tissue samples were immediately immersed in 4% neutral buffered formalin.

### RNA and antibody detection

A sample volume of 140 μl of serum, bile and faecal filtrates were extracted manually with the QIAmp® Viral RNA Mini Kit (QIAGEN GmbH, Hilden Germany) following the manufactures instructions and eluated in 50 μl buffer. Manual RNA extraction from tissue samples was performed with the RNEasy® Mini Kits (QIAGEN GmbH, Hilden Germany). For this purpose 10 mg of a tissue sample was homogenized in 600 μl RLT buffer using TissueLyser II ® (Qiagen). After centrifugation the supernatant was used for RNA extraction according to manufacturer’s instructions. A heterologous internal control [63] was added to each extraction sample. Obtained RNA was stored at − 80 °C until further use.

To monitor the course of the infection a diagnostic quantitative real-time RT-PCR (RT-qPCR) targeting a fragment of ORF3 was performed. Primer, probes and protocol were used as previously reported [23, 39]. Each reaction containing 25 μL had a final primer concentration of 0.8 μM and of 0.1 μM probe and 5 μl RNA. RT-qPCR was carried out using the Quanti Tect Probe RT-PCR Kit (QIAGEN GmbH). The CFX96™Real-Time System (Bio-Rad Laboratories GmbH, Munich, Germany) was set to 50 °C for 30 min for reverse transcription followed by denaturation/activation 95 °C for 15 min. DNA amplification was performed in 45 cycles consisting each of 95 °C for 10 s, 55 °C for 25 s and 72 °C for 25 s in immediate succession. The quantification of RNA (HEV copies/μl RNA) was performed by a standard curve based on serial dilutions of a HEV standard, which was included in each qRT-PCR run (Additional file 1). The copy number of HEV standards was calculated by a synthetic calibrator which consists of the qRT-PCR amplicon (81 nucleotides) and a T7-Promotor sequence at the 5′-end to allow in vitro transcription [39]. International Units/ml (IU) were calculated from the WHO International standard for HEV. This standard was provided by the Paul-Ehrlich-Institut (PEI), Langen, Germany (PEI code 6329/10). All RT-qPCR results are given in HEV copies/μl RNA (originating from 50 μl elution volume). The corresponding cop/ml (originating from 140 μl fluid sample) were calculated by conversion factor of 357, 14 (elution volume divided by fluid sample volume) and is depicted in Additional file 2. The limit of detection of about 1 cop/μl is reached at ct-values of ~ 35. The limit of quantification corresponds to the 10^− 4^ HEV standard at 7 copies/μl. Viral loads below the lowest HEV standard were retested by an alternative HEV specific qRT-PCR [64].

To monitor the immunological responses, the serum samples were tested with two commercially available HEV ELISA kits. One was the species independent HEV-Ab ELISA (AXIOM, Bürstadt, Germany) [23] detecting total serum anti-HEV-antibodies. The other was the porcine specific Priocheck HEV Ab porcine ELISA (Mikrogen GmbH, Neuried, Germany), which is specific for serum Anti-HEV-IgG. Both were carried out and interpreted as described by the manufacturer.

### Sequencing

To monitor and verify the identity of the inoculated compared to the replicated/excreted virus genome a 348 nucleotide long partial sequence of the hypervariable region (HVR) was recovered from corresponding faecal samples. SYBR Green-RT-qPCR followed by a SYBR Green nested PCR both with melting curve analysis was performed as described [39]. The resulting cDNA was sequenced by a commercial provider (Eurofins Genomics GmbH, Ebersberg, Germany).

## Additional files

Additional file 1: Amplification curve of HEV specific qRT-PCR. A) Amplification curve of HEV specific qRT-PCR targeting the HEV standard (red line) and the RNA extracts from the inocula (blue line). B) Standard curves were obtained by Ct values plotted against the log of starting quantity. C) Obtained Ct values and determined copy numbers (DOCX 219 kb)

Additional file 2: Determination of the conversion factor from copies per μl RNA to IU of the WHO standard (DOCX 16 kb)

Additional file 3: Calculation of the Infectivity titer of the Liver Used for Inoculation (DOCX 16 kb)

Additional file 4: Results of the RT-qPCR from serum and the porcine IgG HEV-Ab ELISA; The graphs display the individual curves of viral RNA in serum and the detection of Anti-HEV-IgG antibodies in correlation to days after infection (DOCX 462 kb)

Additional file 5: Alignment of the hypervariable region. Alignment done with Geneious version 10.2 created by Biomatters. Available from https://www.geneious.com (DOCX 244 kb)

## Abbreviations

Ab: Antibody
dpi: Days post inoculation
ELISA: Enzyme-linked immunosorbent assay
f: Female
gt: Genotype
HEV: Hepatitis E Virus
HVR: Hypervariable region
i.v.: Intravenous
Ig: Immunglobulin
IU: International Unit
m: Male
PBS: Phosphate buffered saline
PCR: Polymerace chain reaction
PCV2: Porcine circovirus 2
PRRSV: Porcine reproductive and respiratory syndrome virus
RNA: Ribonucleic acid
