# Supplementary material for: High sensitivity of domestic pigs to intravenous infection with HEV
Source: BMC Vet Res. 2018 Dec 4;14:381. doi: 10.1186/s12917-018-1713-8 (PMC6278151; doi:10.1186/s12917-018-1713-8)
Supplement: Supplementary file 1 — Amplification curve of HEV specific qRT-PCR. A) Amplification curve of HEV specific qRT-PCR targeting the HEV standard (red line) and the RNA extracts from the inocula (blue line). B) Standard curves were obtained by Ct values plotted against the log of starting quantity. C) Obtained Ct values and determined copy numbers (DOCX 219 kb) [file 12917_2018_1713_MOESM1_ESM.docx]

**Additional file 1:** Amplification curve of HEV specific qRT-PCR


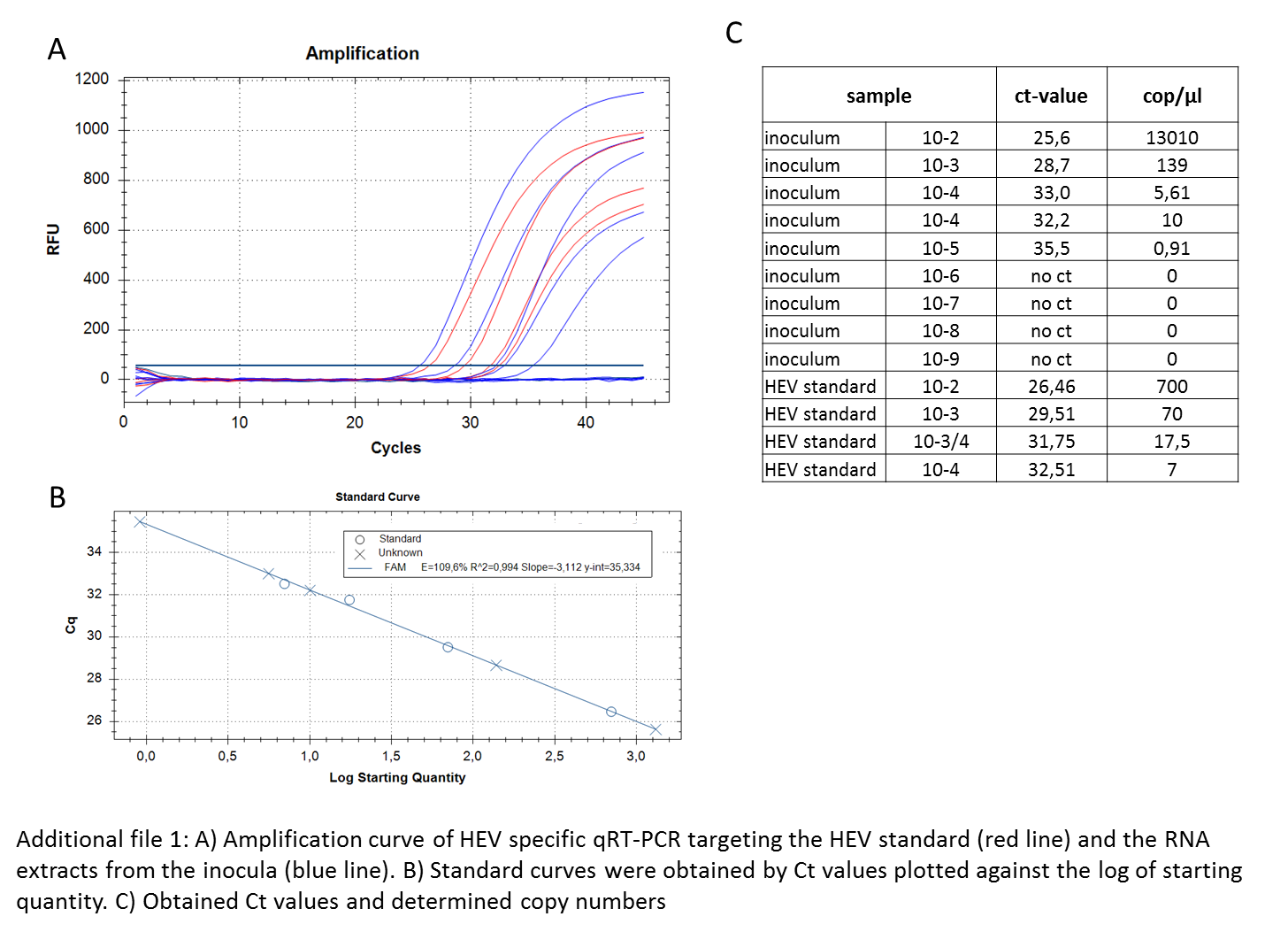


A) Amplification curve of HEV specific qRT-PCR targeting the HEV standard (red line) and the RNA extracts from the inocula (blue line). B) Standard curves were obtained by Ct values plotted against the log of starting quantity. C) Obtained Ct values and determined copy numbers
