# Supplementary material for: High sensitivity of domestic pigs to intravenous infection with HEV
Source: BMC Vet Res. 2018 Dec 4;14:381. doi: 10.1186/s12917-018-1713-8 (PMC6278151; doi:10.1186/s12917-018-1713-8)
Supplement: Supplementary file 2 — Determination of the conversion factor from copies per μl RNA to IU of the WHO standard (DOCX 16 kb) [file 12917_2018_1713_MOESM2_ESM.docx]

**Additional file 2: Determination of the conversion factor from copies per µl RNA to IU of the WHO standard**

|  |  | Standards | Dilution | Copy number (cop/µL)^1^ | Copy number (cop/mL)^2^ | C_t_ Mean | Inernational Units (IU/ml) | International Units (IU/cop) | | |
| --- | --- | --- | --- | --- | --- | --- | --- | --- | --- | --- |
|  | Assay |  |  |  |  |  |  |  |  |  |
|  |  |  |  |  |  |  |  | indiviual values | mean values | total mean value |
|  | Assay 1 | 10^-2#^ | 1,00E-02 | 700 | 250 000 | 27,11 | 759 568 | 3,04 | **2,61 ±0,51** | **3,68 ±0,15** |
|  |  | 10^-3^ | 1,00E-03 | 70 | 25 000 | 29,57 | 80 141 | 3,21 |  |  |
|  |  | 10^-3/4^ | 2,50E-04 | 17,5 | 6 250 | 31,4 | 14 981 | 2,4 |  |  |
|  |  | 10^-4^ | 1,00E-04 | 7 | 2 500 | 32,71 | 4 489 | 1,8 |  |  |
|  |  | WHO 10^0^ |  |  |  | 28,43 | 250 000 |  |  |  |
|  |  | WHO 10^-1^ |  |  |  | 30,71 | 25 000 |  |  |  |
|  |  | WHO 10^-2^ |  |  |  | 33,29 | 2 500 |  |  |  |
|  |  | WHO 10^-3^ |  |  |  | 36,11 | 250 |  |  |  |
|  | Assay 2 | 10^-2#^ | 1,00E-02 | 700 | 250 000 | 28,01 | 1415 676 | 5,66 | **4,76 ±0,82** |  |
|  |  | 10^-3^ | 1,00E-03 | 70 | 25 000 | 31,14 | 137 296 | 5,49 |  |  |
|  |  | 10^-3/4^ | 2,50E-04 | 17,5 | 6 250 | 33,31 | 27 192L | 4,35 |  |  |
|  |  | 10^-4^ | 1,00E-04 | 7 | 2 500 | 34,83 | 8 819 | 3,53 |  |  |
|  |  | WHO 10^0^ |  |  |  | 30,52 | 250 000 |  |  |  |
|  |  | WHO 10^-1^ |  |  |  | 33,03 | 25 000 |  |  |  |
|  |  | WHO 10^-2^ |  |  |  | 36,78 | 2 500 |  |  |  |
|  |  | WHO 10^-3^ |  |  |  | 39,49 | 250 |  |  |  |

^1^ originating from 50µl elution volume

^2^ originating from 140µl fluid samples. Conversion factor 357,14µl/ml
