# Supplementary material for: High sensitivity of domestic pigs to intravenous infection with HEV
Source: BMC Vet Res. 2018 Dec 4;14:381. doi: 10.1186/s12917-018-1713-8 (PMC6278151; doi:10.1186/s12917-018-1713-8)
Supplement: Supplementary file 3 — Calculation of the Infectivity titer of the Liver Used for Inoculation (DOCX 16 kb) [file 12917_2018_1713_MOESM3_ESM.docx]

# Additional file 3

**Calculation of the Infectivity titer of the Liver Used for Inoculation**

| log_10_ dilution | infected | Inoculated |
| --- | --- | --- |
| -2 | 4 | 4 |
| -3 | 4 | 4 |
| -4 | 7 | 8 |
| -5 | 4 | 4 |
| -6 | 2 | 4 |
| -7 | 1 | 4 |
|  |  |  |

The 50% endpoint dilution (ID_50_) of the liver tissue was calculated using the Spearman-Kärber method:

log_10_ ID_50_/ml = a + d/2 - d Σ pi + logV

a: log_10_ of smallest dose where all animals were infected (here: -3)

d: log_10_ of dilution factor (here: 1 because of a 10-fold dilution series)

p_i_: reactivity rate per dilution step i

V: volume inoculated (here: 2ml)

Based on the rate of infected animals the following log_10_ ID_50_/ml was calculated:

-3 + 0.5 –(4/4+7/8+4/4+2/4+1/4) + log 2ml= -5.8

The statistical titer is then the antilogarithm of the negative log_10_ ID_50_/ml

| Titer=10 ^5.8^ ID_50_/ml = 6.3x 10^5^ ID_50_/ml |
| --- |

Based on the Poisson distribution the relation between infectious units and the ID_50_/ml is ln 2 ~ 0.7. Therefore, 6.3x 10^5^ ID_50_/ml correspond to 4.4x 10^5^ infectious units/ml.

**Calculation of the Ratio IU HEV RNA per infectious unit**

In Table 2 the HEV RNA content was determined to be 3.4x 10^8^ IU HEV RNA per 2ml original liver tissue, i.e. 1.7x 10^8^ IU/ml. Therefore, the relation of HEV RNA to infectious units in this infectivity model is as follows:

1.7x 10^8^ IU/ml / 4.4x 10^5^ infectious units/ml = 773 IU HEV RNA per 1 infectious unit.
