# Supplementary material for: High sensitivity of domestic pigs to intravenous infection with HEV
Source: BMC Vet Res. 2018 Dec 4;14:381. doi: 10.1186/s12917-018-1713-8 (PMC6278151; doi:10.1186/s12917-018-1713-8)
Supplement: Supplementary file 4 — Results of the RT-qPCR from serum and the porcine IgG HEV-Ab ELISA; The graphs display the individual curves of viral RNA in serum and the detection of Anti-HEV-IgG antibodies in correlation to days after infection (DOCX 462 kb) [file 12917_2018_1713_MOESM4_ESM.docx]

Additional file 4


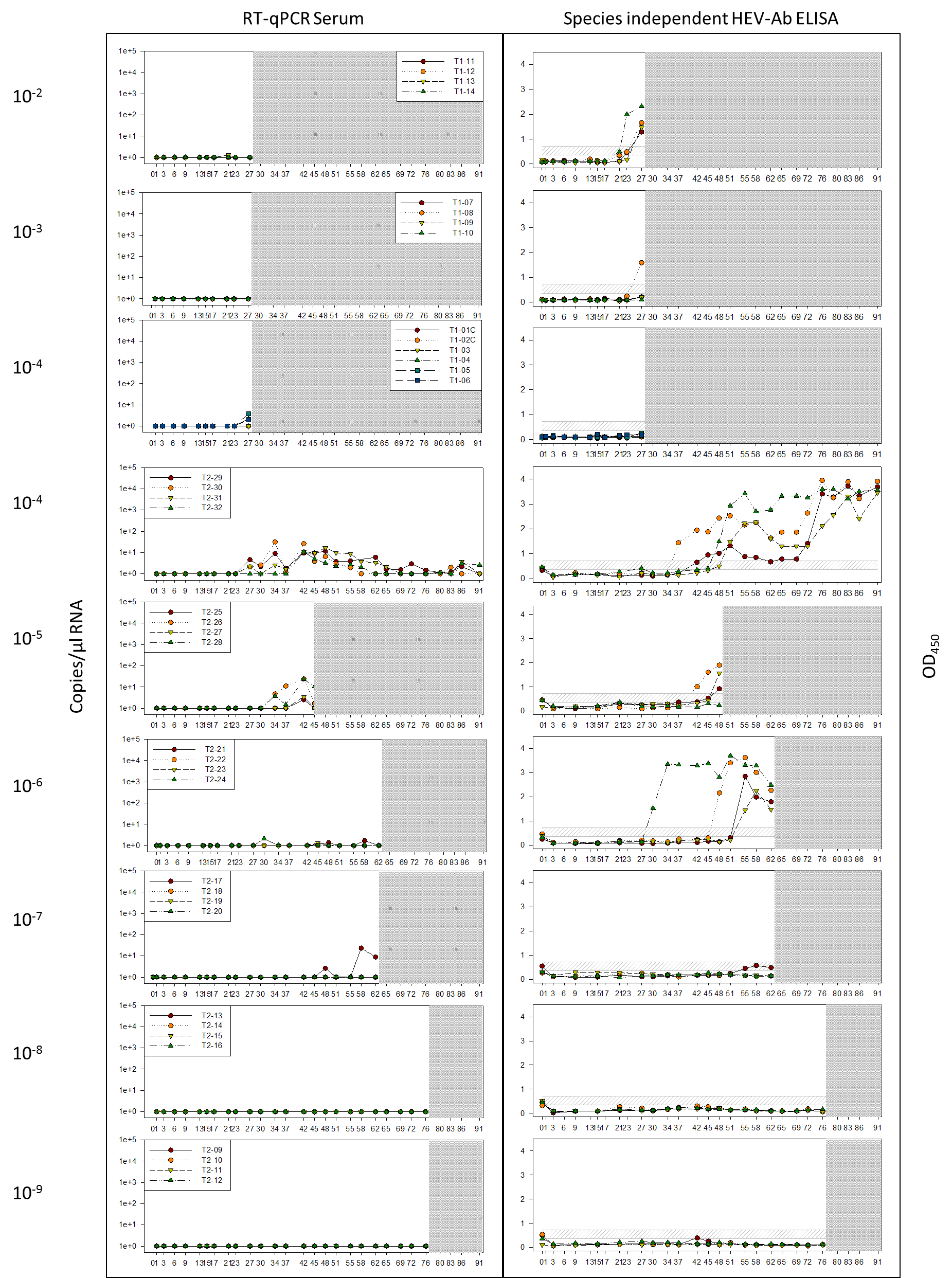
Additional file 4: Results of the RT-qPCR from serum and the porcine IgG HEV-Ab ELISA; the graphs display the individual curves of viral RNA in serum and the detection of Anti-HEV-IgG antibodies in correlation to days after infection
