# Supplementary material for: High sensitivity of domestic pigs to intravenous infection with HEV
Source: BMC Vet Res. 2018 Dec 4;14:381. doi: 10.1186/s12917-018-1713-8 (PMC6278151; doi:10.1186/s12917-018-1713-8)
Supplement: Supplementary file 5 — Alignment of the hypervariable region. Alignment done with Geneious version 10.2 created by Biomatters. Available from https://www.geneious.com (DOCX 244 kb) [file 12917_2018_1713_MOESM5_ESM.docx]

# Additional file 5: Alignment of the hypervariable region


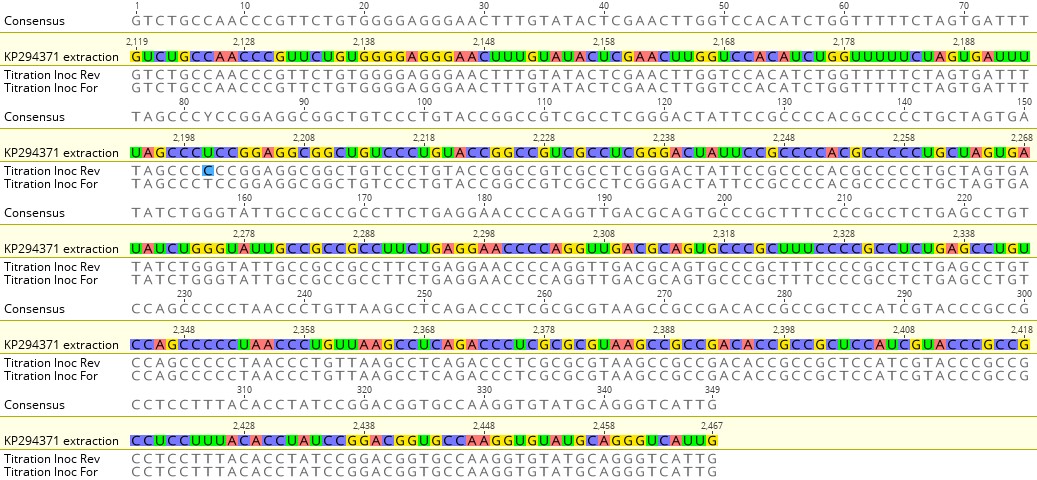


Alignment done with Geneious version 10.2 created by Biomatters. Available from https://www.geneious.com
